# Two Different Mismatches: Integrating the Developmental and the Evolutionary-Mismatch Hypothesis

**DOI:** 10.1177/17456916221078318

**Published:** 2022-07-14

**Authors:** Marèn Hoogland, Annemie Ploeger

**Affiliations:** Department of Psychology, University of Amsterdam

**Keywords:** evolutionary mismatch, developmental mismatch, differential susceptibility, origins of disease, origins of psychopathology, origins of depression

## Abstract

Evolutionary psychology aims to understand the origins of the human mind, including disease. Several theories about the origins of disease have been proposed. One concerns a developmental mismatch—a mismatch might occur at the individual level between the environment experienced during childhood and the environment the adult finds herself in, possibly resulting in disease. A second theory concerns the idea of an evolutionary mismatch—humans are adapted to ancestral conditions so they might now experience a mismatch with their modern environment, possibly resulting in disease. A third theory—differential susceptibility—outlines how genetic and epigenetic differences influence the extent to which humans are susceptible to rearing, including positive and negative experiences. Because of these differences, some individuals are more prone to develop disease than others. We review empirical studies that substantiate these theories and argue that an overarching theory that integrates these three lines into one provides a more accurate understanding of disease from an evolutionary perspective.

Two different mismatch hypotheses have been proposed in the literature, one concerning developmental mismatches in one’s individual lifetime (e.g., Barker, 2004; Bateson et al., 2004; Gluckman et al., 2008; Schmidt, 2011) and one concerning mismatches on an evolutionary timescale (e.g., Gluckman & Hanson, 2004; Li et al., 2018). The developmental-mismatch hypothesis states that early experiences shape the brain and behavior; major differences in stressors between the early years of development and later life stages are mismatches that can cause disease (both physically, e.g., cardiovascular, and mentally, e.g., depression). For example, individuals that experienced a safe and harmonious childhood tend to have a hard time dealing with major stressors later in life. On the other hand, individuals that grew up in an unsafe and stressful environment tend to be prepared to deal with stress. The developmental-mismatch hypothesis is often contrasted with the cumulative-stress hypothesis, which states that ongoing stress during one’s life results in an increased risk to develop disease (e.g., Nederhof & Schmidt, 2012).

The evolutionary-mismatch hypothesis states that differences in stressors between the environment in which humans evolved and the current environment are mismatches that can cause disease. Up until 10,000 years ago, humans lived a nomadic lifestyle as hunter-gatherers, with different stressors from the ones that people experience nowadays in modern environments. Examples of evolutionary mismatches are different food patterns (e.g., Logan & Jacka, 2014), different sleep patterns (e.g., Samson et al., 2017), lack of exercise (e.g., Lieberman, 2015), lack of natural daylight (e.g., Veitch & McColl, 2001), lack of green environments (e.g., Joye & van den Berg, 2011), lack of social cohesion (e.g., Hawkley & Capitanio, 2015), and the current information overload (e.g., Blease, 2015). Each of these individual mismatches and the sum of several mismatches in one’s life result in an increased risk to develop disease.

Given the substantial support for both hypotheses (briefly reviewed below), it follows naturally that the two hypotheses need to be integrated to provide a full account of the development of disease. In addition, theoretically, it is also necessary to take into account both developmental and evolutionary processes to get a better understanding of the arising of variation in the population. Evolutionary-developmental biology stresses the importance of the interaction of evolutionary and developmental processes, which leads to research questions such as how a novel variation arises. Is evolution biased by developmental constraints? Which mechanisms facilitate or constrain evolutionary change? How do these mechanisms relate to plasticity, robustness, and epigenetic causation of phenotypes (e.g., Ploeger & Galis, 2011)? The interplay between developmental and evolutionary processes in the arising of diseases has not been stressed in the literature very much. This article is meant to fill this gap.

Related to this, it is known that there are substantial individual differences in reaction toward stressors. These differences have been described in the literature under different names, for example, *differential susceptibility* (e.g., Belsky & Pluess, 2016), *programming sensitivity* (e.g., Nederhof & Schmidt, 2012), *biological sensitivity to context* (e.g., Ellis & Boyce, 2008), *undirected susceptibility to change* (e.g., Branchi, 2011), *sensory-processing sensitivity* (e.g., Aron & Aron, 1997), and *developmental plasticity* (e.g., Gluckman et al., 2009). All these different accounts assume individual differences in susceptibility to environmental circumstances. The aim of the present article is to integrate the developmental- and the evolutionary-mismatch hypotheses about the risk to develop disease, combined with an account of individual differences in differential susceptibility. We first briefly review the evidence for the developmental-mismatch hypothesis, the evolutionary-mismatch hypothesis, and differential susceptibility. Given the breadth of these three topics, our aim is not to provide a systematic review but rather an integrative one that leads to a new proposal to unify these different approaches to develop an overarching model to explain the development of disease.

## Support for the Developmental-Mismatch Hypothesis

**Fig. 1.**
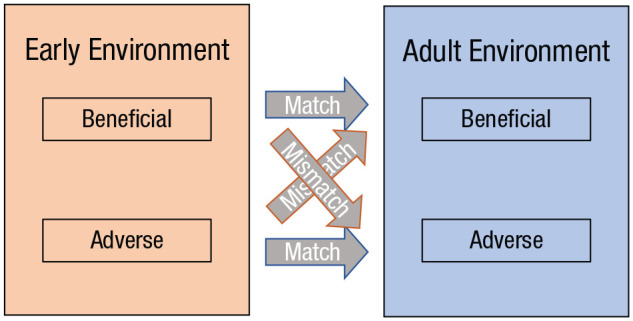
The developmental-mismatch hypothesis. When there is a match between the early environment and adulthood (blue arrows), a decreased risk for disease is expected. When there is a mismatch between the two periods (red arrows), an increased risk for disease is expected (Schmidt, 2011).

The developmental-mismatch hypothesis is outlined in Figure 1. Most empirical studies are with animal models, which allow for an experimental design.

For example, Santarelli et al. (2014) tested the developmental-mismatch hypothesis by rearing female mice under stressful or enriched conditions and subdivided both groups during adulthood into groups with aversive versus positive environments, which resulted in matched and mismatched individuals. The matched individuals displayed more social, less anxious, and stress-coping behaviors compared with the mismatched individuals. In a subsequent study with male mice and a similar design, it was found that early life stress plus adult life stress protects against neuroendocrine, behavioral, and molecular effects of early life stress (Santarelli et al., 2017). Both studies are consistent with the developmental-mismatch hypothesis.

Another experimental study examined a mismatch effect of early and adult stress on hippocampal-dependent memory in rats (Zalosnik et al., 2014). A mismatch group (no stress in early life combined with adult chronic stress) showed poor hippocampal-memory performance compared with the matched groups. Another study in female rats compared individuals exposed to perinatal stress and unpredictable chronic mild stress during adulthood with individuals with equal levels of perinatal stress but normal levels of stress during adulthood (Van Camp et al., 2018). The group with adult stress displayed a reduced body weight gain, showed improved behavioral performance in response to novelty and motivational tests, and exhibited increased estradiol levels with an unmodified profile of a regular estrous cycle in stress response and risk-taking behavior compared with the group with normal stress levels during adulthood.

A few studies have tested the developmental-mismatch hypothesis in humans. For example, Sandman et al. (2012) tested whether the congruence of maternal depression or lack thereof during the prenatal and postnatal periods affects infant development differently from infants who were exposed to either prenatal or postnatal maternal depression. They showed increased motor and mental development during the first year in the infants from congruent mothers compared with infants who were exposed to adversity only prenatally or postnatally. In another study, 2,230 individuals were grouped according to their attention style, defined as a shifter (diverging attention over different spots), a sustainer (keeping attention at a single spot), or both (i.e., being nonspecialized; Nederhof et al., 2014). Early life stress and recent levels of stress were measured and related to depression scores in adulthood. Evidence for the mismatch hypothesis was found in the sustainer group, and evidence for the cumulative-stress hypothesis was found in the nonspecialized group. The third group, the shifters, seemed insensitive to stress in terms of later development of depression.

However, in a large study with participants whose early life adversities and recent life adversities were measured and related to current symptoms of anxiety and depression and brain morphology, Kuhn et al. (2016) found no evidence for a statistical interaction between early and recent adversities, which supports the cumulative-stress hypothesis. A study on young individuals with mental-health problems tested the cumulative-stress and developmental-mismatch hypotheses in relation to clinical outcomes and structural and functional brain imaging (Paquola et al., 2017). On the basis of data about childhood abuse and recent stress, two matched and two mismatched groups were formed. The relation between childhood abuse and recent stress on severity of psychiatric symptoms and structure and function of social and cognitive brain regions was compared between the groups. For all groups, lifetime stress was related to severity of psychiatric symptoms, which supports the cumulative-stress hypothesis. The neuroimaging data, however, showed reduced left hippocampal volume, reduced anterior cingulate cortex (ACC)-ventrolateral prefrontal cortex resting-state functional connectivity (rsFC) and greater ACC-hippocampus rsFC in the mismatched groups compared with the matched groups. These neuroimaging data may provide the neural basis for decreased social behavior and hippocampal memory (Ricon et al., 2012; Santarelli et al., 2014), and worse metacognitive abilities (Baird et al., 2013) and thus support the developmental-mismatch hypothesis.

To conclude, the experimental evidence based on animal studies for the developmental-mismatch hypothesis is strong. However, the correlational evidence in human studies is mixed. This mixed evidence might be due to confounding variables and large individual differences in response to stressors.

## Support for the Evolutionary-Mismatch Hypothesis

Advocates of the evolutionary-mismatch hypothesis have argued that human beings evolved in an entirely different environment from the one in which modern people currently live, which has resulted in a mismatch (e.g., Gluckman & Hanson, 2006; Li et al., 2018). Because the genetic makeup and the brain are not well adapted to current modern environments, humans are prone to develop disease. Support for this hypothesis comes from several sources.

First, there is a positive correlation between a modern lifestyle and all kinds of physical diseases (e.g., Bhatnagar, 2017; Thorburn et al., 2014). For example, diseases such as obesity, hypertension, type 2 diabetes, coronary heart disease, epithelial cell cancers, osteoporosis, and autoimmune disease are hardly present in hunter-gatherers and other nonmodernized populations (for a review, see Carrera-Bastos et al., 2011). An increase in these diseases is observed when people adopt a modern lifestyle, including the consumption of processed meat and sugar-sweetened beverages (e.g., Micha et al., 2017); a lack of sunlight and the associated vitamin-D deficiency (e.g., Mozos & Marginean, 2015); sleep patterns that deviate from the natural circadian rhythm (e.g., Kohansieh & Makaryus, 2015); chronic stress, as opposed to acute stress (e.g., Lagraauw et al., 2015); too much sedentary time (e.g., Biswas et al., 2015); a lack of physical activity (e.g., Kyu et al., 2016); and exposure to human-made pollutants (e.g., Liu et al., 2015).

Second, a modern lifestyle is positively correlated with mental disorders. For example, disorders such as depression are associated with a lack of exercise (e.g., Hiles et al., 2017), the consumption of too little nonprocessed food (e.g., Sanhueza et al., 2013), a lack of sleep (e.g., Luca et al., 2013), a vitamin-D deficiency (e.g., Anglin et al., 2013), cigarette smoking (e.g., Fluharty et al., 2017), exposure to social media (e.g., Keles et al., 2020), perfectionism (e.g., Limburg et al., 2017), and chronic stress (e.g., McGonagle & Kessler, 1990). However, these correlational studies do not tell whether adopting a modern lifestyle causes depression or whether people with depression adopt an unhealthy lifestyle. Evidence for a causal relationship comes from experimental studies in which lifestyle factors have been manipulated to examine the effects on depression.

So the third piece of support for the evolutionary-mismatch hypothesis comes from randomized control trials in which the effects of lifestyle treatments for different mental disorders, such as depression, have been studied. Positive effects of changing lifestyle factors on disorders, such as depression, have been found, including the increase of exercise (e.g., Kvam et al., 2016; Schuch et al., 2016), change of diet (e.g., Jacka et al., 2017), nonpharmacological interventions for sleep problems (e.g., Gee et al., 2019), vitamin-D supplementation and augmentation (e.g., Parker et al., 2017), and interventions that target chronic stress, such as mindfulness (e.g., Kuyken et al., 2016) and yoga (e.g., Cramer et al., 2017).

Fourth, there is preliminary evidence that the prevalence of mental disorders is higher among modern societies compared with nonindustrial societies. For example, Colla et al. (2006) compared the prevalence of depression in women living in four different locations, rural Nigeria, urban Nigeria, rural Canada, and urban United States, by assessing lifetime depression using interview items, based on the *Diagnostic and Statistical Manual of Mental Disorders*, that were common to all four samples. The prevalence was lowest among women in rural Nigeria and highest among women in urban United States. In addition, several studies reported an increase in depression in China in response to the rapid modernization that occurred between 1990 and 2010 (for a review, see Sun & Ryder, 2016). Similar findings have been reported in India (e.g., Chandra et al., 2018). However, caution is necessary in interpreting these data because depression may be expressed differently in different cultures, making it hard to assess the prevalence of depression cross-culturally (e.g., Kirmayer et al., 2017).

In sum, the association between a modern lifestyle and the risk of developing diseases and mental disorders has been frequently reported in the scientific literature. Experimental studies suggest that this relationship is causal. So we need to take into account modern lifestyle factors as stressors in explanatory models of disease.

## Support for Differential Susceptibility and Its Underlying Mechanisms

Large individual differences exist in the reaction to stressors (Cohen & Hamrick, 2003). It is argued that these individual differences are adaptive in evolutionary terms (Ellis & Del Giudice, 2019), and this has been called *adaptive developmental plasticity* (Gluckman et al., 2009) or *differential susceptibility* (e.g., Ellis et al., 2011). Differential susceptibility describes how individuals differ in how much they adapt to the environment, in other words, how sensitive they are to their environment (Boyce & Ellis, 2005; Branchi, 2011; Nederhof & Schmidt, 2012). Highly susceptible children who grow up in harsh conditions and highly susceptible children who grow up under very supportive conditions develop sustained changes, whereas these changes are less pronounced in less susceptible children in the same circumstances. The notion that the susceptibility can have both “negative” and “positive” outcomes, depending on the developmental context, is what separates the model of differential susceptibility from the previously dominant paradigm, the diathesis-stress model (Ellis et al., 2011).

The underlying traits that constitute high susceptibility are, in behavioral terms, negative emotionality and high sensitivity (Belsky & Pluess, 2009, 2013). In addition to these traits, certain so-called plasticity alleles have been identified, for example, the short allele of 5HTTLPR, the A1 allele of DRD2, the 7R allele of DRD4, the 2R/3R alleles of MAOA, and the 10R allele of DAT1. There is mixed evidence for the interaction effect of one of these specific alleles and a range of environmental factors on developmental outcomes, including disease; however, the cumulative effect of several of these genes is significant (e.g., Belsky & Beaver, 2011; Keers et al., 2016).

The question remains what kind of mechanisms lay behind the interaction between specific alleles and environmental factors. The most likely candidates are epigenetic mechanisms (i.e., mechanisms that do not change the DNA sequence but influence the expression of alleles). Examples of such mechanisms are histone modification and DNA methylation. Epigenetic mechanisms are a source of environmentally driven plasticity and provide the basis of the interaction effect between genes (G) and environment (E; e.g., Meaney, 2010).

In sum, there is empirical support for the concept of differential susceptibility. How can this evidence be integrated with the previously discussed hypotheses?

## Integrating the Developmental-Mismatch Hypothesis, the Cumulative-Stress Hypothesis, and Differential Susceptibility

There is support for the developmental-mismatch hypothesis and differential susceptibility, and evidence for the cumulative-stress hypothesis has been reported as well. The integration of the cumulative-stress hypothesis with the developmental-mismatch hypothesis and individual differences in susceptibility was first proposed by Nederhof and Schmidt (2012). The model describes how either the cumulative-stress hypothesis or the developmental-mismatch hypothesis applies to individuals, depending on their differential susceptibility and their level of early life stress. It is proposed that individuals with low susceptibility will suffer from the accumulation of stress throughout their lives. Although these individuals are not very susceptible to environmental stressors, when stressful situations build up, eventually they will develop disease. On the other hand, individuals with high levels of susceptibility will suffer from a mismatch between the levels of stress during early life and adulthood. That is, when these individuals are exposed to stress early in life, they get prepared for stress later in life. However, when these individuals do not experience stress in their youth, they are not prepared (or adapted) and easily get sick when exposed to stressful situations later in life.

To the best of our knowledge, only one study tested this model directly in human adults. Power et al. (2013) collected data about childhood maltreatment (early life stress), recent stressful life events (adult life stress), the variant of the genotype at the 5-HTTLPR region (as an indication of susceptibility), and major depression as outcome measure. They found an interaction effect of childhood maltreatment and having the short allele variant of 5-HTTLPR on major depression. In addition, the odds of major depression were much higher for participants that reported both early and recent life stressors, providing support for the cumulative-stress hypothesis. No evidence was found for the developmental-mismatch hypothesis and differential susceptibility; individuals that experienced childhood maltreatment did not show a decreased risk for developing major depression when exposed to adult life stress, no matter whether they had the short or the long allele variant of 5-HTTLPR. The authors suggested that the developmental-mismatch hypothesis might be true in cases in which the childhood stress was relatively minor, giving room for recovery.

This was examined in a large, prospective, multiwave longitudinal study in which childhood and adult material wealth were chosen as environmental variables (Keers & Pluess, 2017), although this study was not set up to differentiate between the cumulative-stress hypothesis and the developmental-mismatch hypothesis. Interaction of wealth with multiple plasticity genes, including the variants of 5-HTTLPR and DRD2, was assessed; psychological distress was the outcome variable. The results revealed a G × E × E three-way interaction effect showing that individuals with a relatively high number of plasticity genes are more vulnerable to psychological distress when experiencing both childhood and adult poverty. This is consistent with the cumulative-stress hypothesis or possibly for the even stronger effect of stress sensitization (Hammen et al., 2000): People that experienced childhood adversity require less stressful life events as adults to report psychological distress. Similar results have been reported in G × E × E studies on the interaction effect of genotype 5-HTTLPR, severe institutional deprivation, and stressful life events in adolescence on emotional problems (Kumsta et al., 2010); the interaction effect of genotype 5-HTTLPR, childhood abuse, and adult traumatic experience on depressive symptoms in adulthood (Grabe et al., 2012); and the interaction effect of genotypes CRHR1 and 5-HTTLPR, childhood adversity, and recent chronic stress on depressive symptoms at age 20 (Starr et al., 2014).

To conclude, there is support for the combination of differential susceptibility and the cumulative-stress hypothesis, but not combined with the developmental-mismatch hypothesis, to explain the variance in disease in humans. However, there has been only one direct test of the model proposed by Nederhof and Schmidt (2012), and experimental (animal) studies are missing.

## Integrating the Developmental-Mismatch Hypothesis, the Cumulative-Stress Hypothesis, the Evolutionary-Mismatch Hypothesis, and Differential Susceptibility

So far, the evidence seems to be mainly in favor of the integration of the cumulative-stress hypothesis in combination with differential susceptibility to explain the development of disease in humans. That is, some people with low differential susceptibility do not seem to suffer from stressors, whether these occurred during childhood or adulthood, and do not develop disease. Other people with high differential susceptibility tend to suffer most from childhood stressors, and they require relatively few stressors in adult life to develop disease.

However, this is probably not the complete picture. All data with humans are correlational; experimental evidence is hard to gather with humans. Nonhuman animal studies are necessary to get a better understanding of the mechanisms underneath the association found in humans. Animal studies provide support for the developmental-mismatch hypothesis; animals that experienced stress as juveniles and as adults tend to develop less disease than animals that experience a mismatch in conditions. Why would the results for humans and nonhuman animals differ?

One reason could be that the effects of a developmental mismatch in combination with differential susceptibility cannot be detected in correlational studies. Too many confounding variables are present compared with controlled laboratory settings in animal studies. Another reason could be that humans are too different from other animals. For example, humans have a very prolonged juvenile period compared with lab animals, such as mice and rats, and a very complex web of social, cultural, and technological factors that may influence the development of disease. Human culture is so complex and changing so quickly that it is impossible to program children for all the different stressors that they may encounter during life.

A third reason could be that the relationship between potential stressors and the development of disease in humans is complex. For example, a U-shaped relationship between early adversity and stress reactivity was found in a sample of kindergarten children (Shakiba et al., 2020). That is, children who lived in conditions that were classified as either high or low adversity showed high stress reactivity compared with children who lived in average conditions. These kinds of relationships cannot easily be detected with traditional linear statistical methods. So it is too early to rule out the developmental-mismatch hypothesis yet.

What has not been proposed yet is the integration of the cumulative-stress hypothesis, differential susceptibility, the developmental-mismatch hypothesis, and the evolutionary-mismatch hypothesis. As we reviewed above, there is empirical support for the evolutionary-mismatch hypothesis. The different evolutionary-mismatch factors provide a great opportunity to study social, cultural, and technological stressors that have not been examined in past research on the development of disease. So far, research has focused on childhood adversity, such as maltreatment, poverty, and institutionalized deprivation. However, it is possible that these factors are too extreme to be able to recover and be prepared for the confrontation with new potential stressors. Evolutionary-mismatch factors include relatively “normal” stressors, such as exposure to social media, lack of exercise, and the consumption of processed food. When the effects of several of these mismatch factors sum up, it is possible to develop disease. Future research may focus on these factors and study the interaction with differential susceptibility on the development of disease. Longitudinal studies need to unravel whether the exposure to several evolutionary-mismatch factors result in a cumulative effect or a developmental-mismatch effect on disease. Our prediction is, on the one hand, that individuals with low differential susceptibility suffer relatively little from evolutionary-mismatch factors, and when they do, it is only when the factors accumulate (the cumulative-stress hypothesis). On the other hand, individuals with high differential susceptibility suffer more from evolutionary-mismatch factors both when the factors accumulate (because of stress sensitization) and when there is a mismatch between the exposure to these factors in childhood and adulthood (because of an absence of being prepared to deal with the presence or absence of these stressors).

## Conclusion

To create a complete model to explain the variance in the development of disease, it is recommended to integrate the developmental-mismatch hypothesis, the cumulative-stress hypothesis, the evolutionary-mismatch hypothesis, and differential susceptibility. This allows researchers to analyze factors related to the development of disease on three different levels: the level of the genome, the level of development, and the level of the current environment. A thorough analysis of all these levels and a measurement of the degree of evolutionary and developmental mismatch could explain the occurrence of disease.

## Transparency

*Action Editor:* Laura A. King

*Editor:* Laura A. King
